# Design and implementation of elliptical mantle cloaks for polarization decoupling of two tightly spaced interleaved co-frequency patch array antennas

**DOI:** 10.1038/s41598-023-29889-y

**Published:** 2023-02-18

**Authors:** Reza Masoumi, Robab Kazemi, Aly E. Fathy

**Affiliations:** 1 Faculty of Electrical and Computer Engineering, University of Tabriz, 5166616471 Tabriz, Iran; 2 Department of Electrical Engineering and Computer Science, University of Tennessee, Knoxville, 37996 USA

**Keywords:** Electrical and electronic engineering, Techniques and instrumentation

## Abstract

In this paper, we utilized the method of mantle cloaking to decouple/isolate two densely packed interleaved patch array antennas operating at the same frequency but with orthogonal polarizations. To reduce mutual coupling between the adjacent elements, vertical strips, as a type of elliptical mantle cloaks, are located in close proximity to the patches. At the operating frequency of *f*_0_ = 3.7 GHz, the edge-to-edge spacing of the elements of the two interleaved arrays is less than *λ*_0_/80 (1 mm) and the center-to-center spacing of each array element is 0.7 *λ*_0_ (57 mm). The proposed design is implemented using 3D printing technology, and its performance in terms of return loss, efficiency, gain, radiation patterns, and isolation is measured for evaluation. The results show the radiation characteristics of the arrays are perfectly retrieved after cloaking similar to the isolated arrays. Decoupling tightly spaced patch antenna arrays on a single substrate paves the way to achieve miniaturized communication systems with full duplex operation or dual polarization communication.

## Introduction

High decoupling and inter-element isolation of antennas have been achieved by utilizing defected ground structure (DGS)^1–3^, parasitic elements^4–6^, electromagnetic band-gap structures^7^, polarization conversion isolators^8–10^, and metamaterial-based DGSs^11,12^. These techniques, however, have limitations such as large size, multilayer structure, and high manufacturing complexity and cost. Additionally, most of these techniques are applicable to antennas operating at different frequencies and the isolation is achieved through the use of frequency-sensitive filters. Therefore, these methods are not suitable for decoupling co-frequency antennas.

On the other hand, electromagnetic cloaking to provide isolation was first used by Pendry^13^ who utilized metamaterials to hide a metal cylinder from incident waves by using transformation-based cloaking method. Later, other cloaking methods, such as transmission-line cloaking^14–16^ and plasmonic cloaking^17–19^ were introduced and developed for various structures. A common drawback of such aforementioned cloaking techniques is the use of bulky metamaterials, which are challenging to implement and whose thicknesses are often greater than or comparable to the size of the area to be covered. Alternatively, mantle cloaking, that is, cloaking by a surface, was developed in^20^ and due to its light weight, ease of fabrication, low cost, and versatile architecture, it has replaced the previous bulky metamaterial cloaks. The primary use of mantle cloaking was to hide the passive objects from radiating devices, e.g., radars^21,22^ upon reducing or canceling the scattering of the objects.

The scheme was extended to hide larger objects with complicated structures, as well as using multilayer mantle cloaks^23–26^. Meanwhile, polarization-sensitive metasurfaces have been used when surfaces behave differently for TE and TM incident waves^27,28^. But, cloaking of the antennas or active devices becomes more challenging if it affects their impedance matching and radiation characteristics. The cloak structure surrounding an antenna should not only preserve its input impedance and radiation characteristics, but also decouple it from adjacent radiators. Fortunately, success has been reported in cloaking cylindrical and printed monopole^29,30^, and Yagi-Uda antennas^31^. These antennas have a relatively simple structure and low gain, which makes them a poor candidate for new communication systems like those for 5G and 6G mobile networks.

In addition to linear cloaks, nonlinear cloaks made of metasurfaces loaded with nonlinear components, such as diodes and active devices have been introduced that would operate at on/off cloaking mode, according to the input power^32–34^ or the incident wave^35,36^. So far, only simple monopole antennas have been cloaked by nonlinear cloaks. As the antenna structure becomes complex, higher order modes are excited, which impacts scattering, and consequently, it is not possible to hide it by single-layer mantle cloaks^37,38^. Additionally, the loaded active devices have frequency-limited operation. These challenges need to be resolved to make nonlinear cloaking more practical. Here we focus on linear cloaking to decouple two interleaved patch arrays.

In this paper, we investigate the isolation of two tightly spaced interleaved patch antenna arrays, with the same operating frequency and orthogonal polarizations, where we extend the idea of the mantle cloaking method and the scattering cancellation concept to cloak and decouple tightly spaced patches. Given that there is an ever-increasing demand in wireless communication capacity, which requires the placement of dense array antennas in highly compact systems for various applications such as MIMO systems, radar detection, mobile communications, etc. The use of interleaved antenna arrays can reduce the space occupied by two separate arrays by almost half. In this design, the edge-to-edge spacing between the elements of the arrays has been significantly reduced to less than *λ*_0_/80 (1 mm), which is much less than in previously designed arrays. The arrays were manufactured at a low cost using 3D printing technology, and the return loss and decoupling between the elements of the arrays have been significantly improved using the proposed cloaking structure. Additionally, their measured radiation characteristics are almost the same as that of the isolated arrays. Such design is a suitable candidate for the implementation of compact communication antennas.

## Design method

Two patch array antennas were designed independently on 80%-filled polylactic acid (PLA) that is compatible with our lab-3D printing manufacturing process. The PLA has *ε*_r_ = 2.2, tan*δ* = 0.013 and a thickness of *h* = 1.524 mm. Dimensions of coaxially fed rectangular patch antennas were calculated to excite TM_01_ mode at *f*_0_ = 3.7 GHz. The elements of the first array are arranged in H-plane while those for the second array are in the E-plane configuration to obtain orthogonal polarizations. To reduce surface wave effects, we used a thin substrate where ${h \mathord{\left/ {\vphantom {h \lambda }} \right. \kern-0pt} \lambda } < 0.048/\sqrt {\varepsilon_{r} }$^39^.

**Figure 1:**
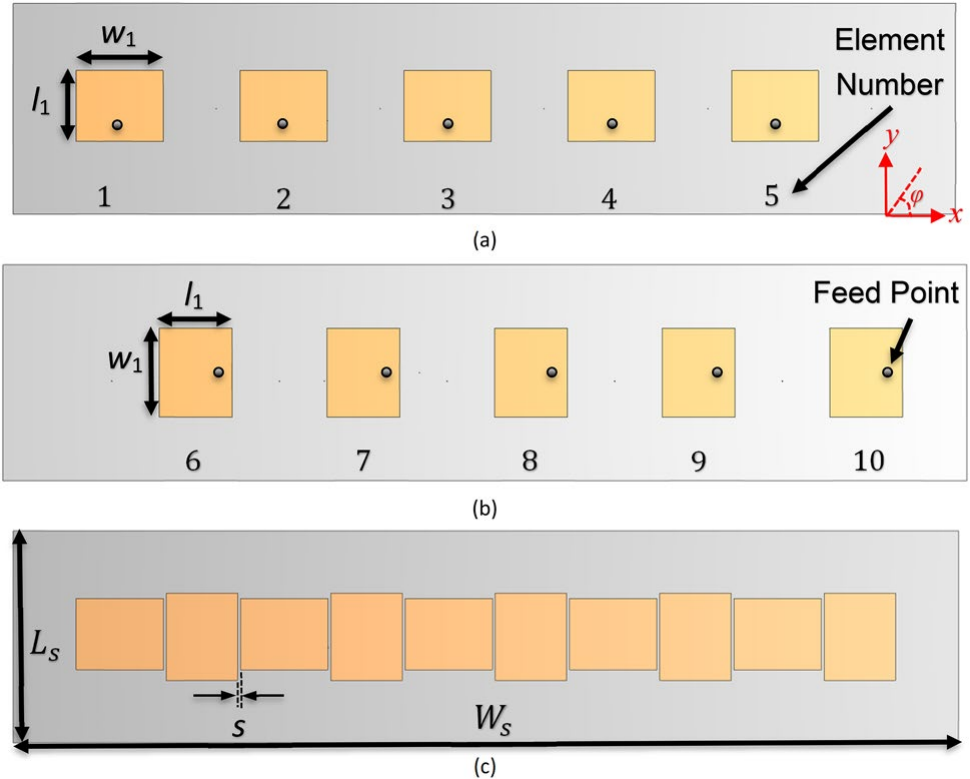
Configurations of the two patch arrays. (**a**) array I in H-plane arrangement, (**b**) array II in E-plane arrangement, (**c**) two tightly spaced interleaved uncloaked arrays.

Two 5×1 linear arrays of patch antennas are shown in Fig. 1a,b for horizontal and vertical polarizations. In this configuration, co-polarization pattern of the array I (elements #1–5) is in the *φ* = 0° plane and that of array II (elements #6–10) is in the *φ* = 90° plane. In the interleaved configuration, Fig. 1c, the edge-to-edge spacing between the adjacent elements is 1 mm (*λ*_0_/80). The optimized dimensions of the arrays are summarized in Table 1.

**Table 1:** Dimensions of the two interleaved patch array antennas.

Parameters	$w_{1}$	$l_{1}$	$W_{s}$	$L_{s}$	$s$
Value (mm)	31.1	25.4	336	75.5	1

Due to the tight spacing between the interleaved patches, each radiating patch induces a significant surface current on the adjacent elements, which acts as a secondary source. The radiation (scattering) from this secondary source affects the impedance matching and radiation characteristics of the primary patch antenna. Hence, in order to eliminate the strong undesired cross-coupling between the interleaved two arrays, an elliptical metasurface mantle cloak is used here over all elements to cancel this unwanted scattering. The orientation of the cloaks covering each patch antenna not only provides the possibility of canceling the unwanted scattering, but also does disturb the radiation characteristics of the cloaked antennas.

Specifically, the coaxially-fed *i*th patch will radiate and will couple to the neighboring *j*th patch. An explicitly tailored elliptical cloak is placed on the *j*th element to cancel its scattering due to the induced surface current due the radiation of the *i*th element at the operating frequency of the *i*th element, which in this case both antennas have the same operating frequency. The ultra-thin metasurface cloak covering the *j*th patch creates an anti-phase surface current with the unwanted induced surface current. This results in a suppression of the induced currents in the cloaked antenna. Therefore, the coupling between the adjacent patches is canceled and does not affect the input impedance and radiation of the elements. Now, only the currents generated by the power supply are involved in the far-field radiation.

### Solving the scattering problem by approximating the patch antenna with an infinite PEC cylinder

Modeling infinitely extended cylinders is conceptually simple because they reduce the 3D scattering problem to a 2D one. However, they do not exist in nature. Therefore, deriving approximate solutions for finite cylindrical structures from the results of infinitely extended structures is the first step toward the analysis of more realistic structures. Here, the patch antenna is modeled by an infinite elliptical cylinder, where the width of the metal patch is the major diameter of the cylinder and the thickness of the patch (tending to zero) is its minor diameter. Therefore, in deriving the analytical formulas, the width of the patch is exact without any approximation. The only approximation for finite case formulation is the finite length of the patch compared to the infinite length of the cylinder. In the following, the effect of this approximation is investigated.

**Figure 2:**
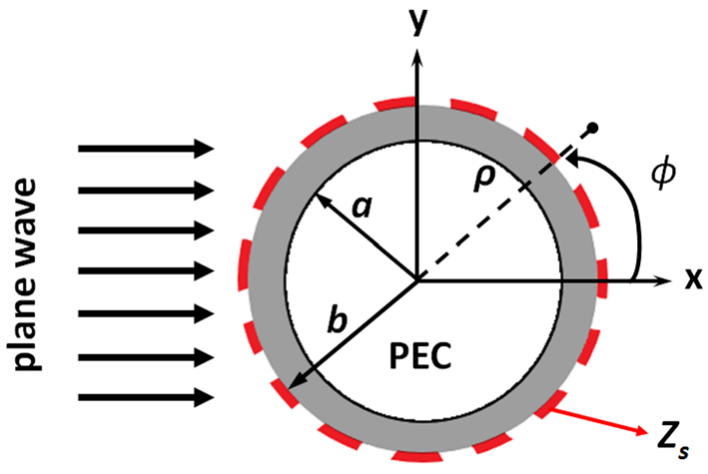
Scattering of a plane wave by a cloaked infinite PEC cylinder.

As is well known, mantle cloaking has so far been used to reduce the scattering of simple passive objects such as spheres and cylinders. Scattering from dipoles/monopoles resembling infinitely long cylinders has been deduced by formulating incident, transmitted and scattered fields. If the cross section of the infinite cylinder is circular, these fields are written benefiting from the cylindrical symmetry of the object^40^. The geometry of the problem is depicted in Fig. 2.

Considering a metallic cylinder covered by a mantle cloak and illuminated by a TM plane wave with an electric field along the *z-axis*, the incident electric and magnetic fields are written in terms of Fourier–Bessel series as:

1 $$E_{z}^{inc} (\rho ,\phi ) = E_{0} \sum\limits_{n = - \infty }^{\infty } {i^{n} J_{n} (k_{0} \rho )e^{in\phi } }$$

2 $$H_{\varphi }^{inc} (\rho ,\varphi ) = \frac{{iE_{0} }}{{\eta_{0} }}\sum\limits_{n = - \infty }^{\infty } {i^{n} J_{n}^{^{\prime}} (k_{0} \rho )e^{in\varphi } }$$

where *E*_0_ is the amplitude of the incident electric field, and *η*_0_ and *k*_0_ are the wave impedance and wavenumber in free space, respectively. The scattered fields in terms of Hankel functions series consisting of outgoing waves are:

3 $$E_{z}^{sc} (\rho ,\phi ) = E_{0} \sum\limits_{n = - \infty }^{\infty } {i^{n} A_{n} H_{n}^{(1)} (k_{0} \rho )e^{in\phi } }$$

4 $$H_{\varphi }^{sc} (\rho ,\varphi ) = \frac{{iE_{0} }}{{\eta_{0} }}\sum\limits_{n = - \infty }^{\infty } {i^{n} A_{n} [H_{n}^{(1)} (k_{0} \rho )]^{^{\prime}} e^{in\varphi } }$$

By applying the boundary conditions at the surface of PEC cylinder ($\rho = a$) and at the interface of the covering dielectric and the metasurface strips ($\rho = b$), the unknown scattering coefficient $A_{n}$ is obtained in terms of Hankel and Bessel functions and effective surface impedance (*Z*_*S*_) of the covering metasurface:

5 $$A_{n} = \frac{{\alpha J_{n}^{^{\prime}} (kb) - \sqrt {\varepsilon_{r} } \beta J_{n} (kb) + i\alpha \eta_{0} J_{n} (kb)/Z_{s} }}{{\sqrt {\varepsilon_{r} } \beta H_{n}^{(1)} (kb) - \alpha [H_{n}^{(1)} (kb)]^{^{\prime}} - i\alpha \eta_{0} H_{n}^{(1)} (kb)/Z_{s} }}$$

where $\alpha = H_{n}^{(2)} (kb)P_{n} + H_{n}^{(1)} (kb)$, $\beta = [H_{n}^{(2)} (kb)]^{^{\prime}} P_{n} + [H_{n}^{(1)} (kb)]^{^{\prime}}$, $P_{n} = - H_{n}^{(1)} (ka)/H_{n}^{(2)} (ka)$. The scattering coefficient, $A_{n}$, determines the total scattering cross section ($\sigma$), which is defined as the total power scattered by a cloaked cylinder to the incident power per unit length of the scatterer:

6 $$\sigma_{total} = \frac{4}{{k_{0} }}\sum\limits_{n = - \infty }^{\infty } {|A_{n} |^{2} }$$

Therefore, if the scattering coefficient $A_{n}$ is zero, the invisibility of the scattering object is achieved. If the scattering object is assumed to be lossless, by tailoring the cloak parameters, the desired $X_{s} = {\text{Im}} (Z_{s} )$ can be determined to achieve near zero $A_{n}$.

If the cross section of the cylinder is elliptical, the analysis of the problem is performed by the method of separation of variables and the well-known Mathieu angular and radial equations. In this case, the incident and scattered electric and magnetic fields are expressed in terms of Mathieu’s odd and even functions in elliptical coordinates^41^, as follows:

7 $$E_{z}^{i} = \sqrt {8\pi } \sum\limits_{n} {j^{ - n} \frac{{J_{pm} (q_{0} ,u,n)}}{{N_{pm} (q_{0} ,n)}}} S_{pm} (q_{0} ,v,n)S_{pm} (q_{0} ,\varphi ,n)$$

8 $$E_{z}^{s} = \sqrt {8\pi } \sum\limits_{n} {j^{ - n} \alpha_{pm}^{(n)} H_{pm}^{(1)} } (q_{0} ,u,n)S_{pm} (q_{0} ,v,n)S_{pm} (q_{0} ,\varphi ,n)$$

where $J_{pm} (q_{0} ,u,n)$ is the radial Mathieu function of the first kind, $S_{pm} (q_{0} ,v,n)$ is the angular Mathieu function, $N_{pm} (q_{0} ,n)$ is the normalization constant, $u$ is the radial distance, $v$ is the angular coordinate, $F$ is the focal point of the elliptic cylinder, $q_{0} = k_{0}^{2} \frac{{F^{2} }}{4}$, $H_{pm}^{(1)} (q_{0} ,u,n)$ is the radial Mathieu function of the third kind indicating the outgoing wave, and $\alpha_{pm}^{(n)}$ are the unknown coefficients.

The Magnetic incident and scattered fields can be determined using Maxwell’s equations. By applying the boundary conditions at the interface of the metal cylinder and the covering cloak, the unknown coefficients, $\alpha_{pm}^{(n)}$, are calculated and the total scattering cross section (SCS) is determined as:

9 $$\sigma_{total} = \frac{1}{2\pi }\int\limits_{0}^{2\pi } {\sigma_{2D} (v)dv}$$

10 $$\frac{{\sigma_{2D} }}{\lambda } =^{ \, } \left|\sum\limits_{n} {\sqrt {8\pi } j^{ - 2n} \alpha_{pm}^{(n)} S_{pm} (q_{0} ,v,n)} S_{pm} (q_{0} ,\varphi ,n)\right|^{2}$$

To cancel the total scattering, the scattering coefficient of each mode,$\alpha_{pm}^{(n)}$, is considered zero, and then the surface reactance required to cancel the scattering of each mode is calculated. In the quasi-static approximation, the surface reactance required to cancel the scattering of the first mode, $X_{opt}$, which is the dominant factor in the scattering of the elliptic cylinder, is obtained in a closed form.

In the illumination with TM mode, the SCS of a finite cylinder of length L (3D) can be relevant to the SCS of an infinite cylinder (2D) as^42^:

11 $$\sigma_{total} = \frac{1}{4\pi }\int {\sigma_{3D} d\Omega = } \frac{1}{4\pi }\iint {\sigma_{3D} }\sin \theta d\theta d\varphi$$

12 $$\sigma_{3D} = \sigma_{2D} \frac{{2L^{2} }}{\lambda }\sin^{2} (\theta_{s} ){\text{sinc}}\left(\frac{{k_{0} L}}{2}(\cos (\theta_{i} ) + \cos (\theta_{s} )\right)$$

where $\theta_{i}$ is the incident angle and $\theta_{s}$ is the scattering angle. Here, the incident angle is 90° (normal incidence).

As it can be seen, the SCS of the finite and infinite cylinders are not the same, but proportional to each other. Since by lowering the SCS of the infinite cylinder (2D case), the SCS of the finite cylinder (3D case) decreases as well, therefore, the $X_{s}$ required to cancel the first scattering mode of the infinite case can also be applied to the finite case. The analytical optimum value of $X_{s}$ to “completely” cancel the scattering of the first mode in the 2D (infinite) case, causes a “partial” reduction of scattering in the 3D (finite) case.

In^43^, the maximum scattering cross sections of both uncloaked and cloaked finite cylinders versus frequency are compared for different values of the length. The cloak was designed for an infinite cylinder, but its parameters are used for a cylinder of finite length. It was shown that the highest cancellation occurred for the longest cylinder, which resembled the infinite case. However, as the cylinder became shorter, the cloak still canceled the scattering of the cylinder to an acceptable degree. The key point is that the maximum scattering cancellation occurs at the same frequency for all values of cylinder length.

In summary, if the cloak is perfectly tailored to reduce the scattering of an infinite cylinder at a specific frequency, reducing its length (moving from the infinite to the finite case) does not change the frequency of minimum scattering. Therefore, to avoid the super-complex scattering equations of the finite elliptic cylinder, it is a routine procedure to calculate the design parameters for the infinite case and carry out a minor numerical optimization to reduce the scattering of the finite case as much as possible^44^. This method is used in^45^ as well, and it is demonstrated that the cloak is effective for the finite case by optimizing the initial parameters obtained from the infinite case equations.

When applying the formulas of a 2D infinite structure to a 3D finite structure, the differences can be summarized as follows:

- The amount of scattering cancellation will be greater for the infinite case than for the finite case.
- At normal illumination, there is minimal difference between the scattering cancellation of infinite and finite cases. However, as the incident angle varies from normal (90°) to tangential (0°), the frequency of minimum scattering and the amount of the scattering cross section in the infinite and finite cases become more different^43,45^.
- There are some peaks and resonances in the scattering diagram of the finite case due to the scattering from its edges. In the proposed structure, these resonances do not disturb the performance of the cloak, as the patch antenna has a narrow bandwidth. However, in wideband cases, they can degrade the cloak performance.

### Closed-form formulations for scattering of patch antennas

In this study, since the scattering in the normal incidence of the electric field is dominated by TM polarization, the TM illumination is used to calculate the optimum $X_{s}$ of the covering mantle cloak. While the incident electric field is normal and the patch antenna is narrowband, the initial values of the cloak parameters obtained from the analytical formulas of the infinite case are appropriate to reduce the scattering of the finite case as well, and “extensive” numerical optimization is not required. Minor optimization by adjusting the parameters by ± 5% around their initial values results in perfect decoupling between adjacent patches and restoration of their radiation characteristics to those of an isolated case. Therefore, the entire design procedure becomes practical and straightforward, and complex calculations to obtain the optimal $X_{s}$ of the finite cylinder, which requires solving the scattering problem by iterative numerical methods^46^, are avoided.

To design the cloak structure to cancel the scattering of the *j*th patch antenna, first, we approximate the planar patch with a metallic elliptic cylinder, where its minor diameter tends to zero. This metal cylinder is concentrically covered with a thin dielectric elliptic cylinder with a relative permittivity of *ε*_*r*1_. This thin dielectric coating acts as a spacer between the patch antenna and the metal metasurface strips of the cloak that will be discussed later. In the quasi-static approximation, at the frequency of $\omega$, the closed-form optimum capacitive reactance required to cancel the surface currents induced on the dielectric-coated metallic elliptic cylinder is^41^:

13 $$X_{opt} = - \, \omega \mu F\frac{{(u_{0} - u_{1} )\cosh u_{0} }}{{1 + \left( {q_{0} (u_{0} - u_{1} ) + q_{1} \left( {u_{1} + \frac{1}{2}Ln(q_{1} )} \right)} \right)\sinh 2u_{0} }}$$

where $q_{0} = k_{0}^{2} F^{2} /4$, $q_{1} = k_{1}^{2} F^{2} /4$, $k_{0}$ and $k_{1}$ are the wavenumbers in free space and dielectric material, respectively, $F$ is the focal point of the concentric cylinders, $\mu$ is the permeability of the dielectric material,$u_{0} = \tanh^{ - 1} \left( {\frac{{b_{0} }}{{a_{0} }}} \right)$, $u_{1} = \tanh^{ - 1} \left( {\frac{{b_{1} }}{{a_{1} }}} \right)$, $a_{0}$ and $b_{0}$ are the major and minor diameters of the dielectric elliptic cylinder, and $a_{1}$ and $b_{1}$ are the major and minor diameters of the metallic elliptic cylinder, respectively. Optimum reduction of the scattering from the dielectric-coated metallic elliptic cylinder is achieved by placing the focal points of the metasurface elliptical cloak at the edges of the planar patch^41^, i.e.:

14 $$a_{0}^{2} = b_{0}^{2} + a_{1}^{2}$$

**Figure 3:**
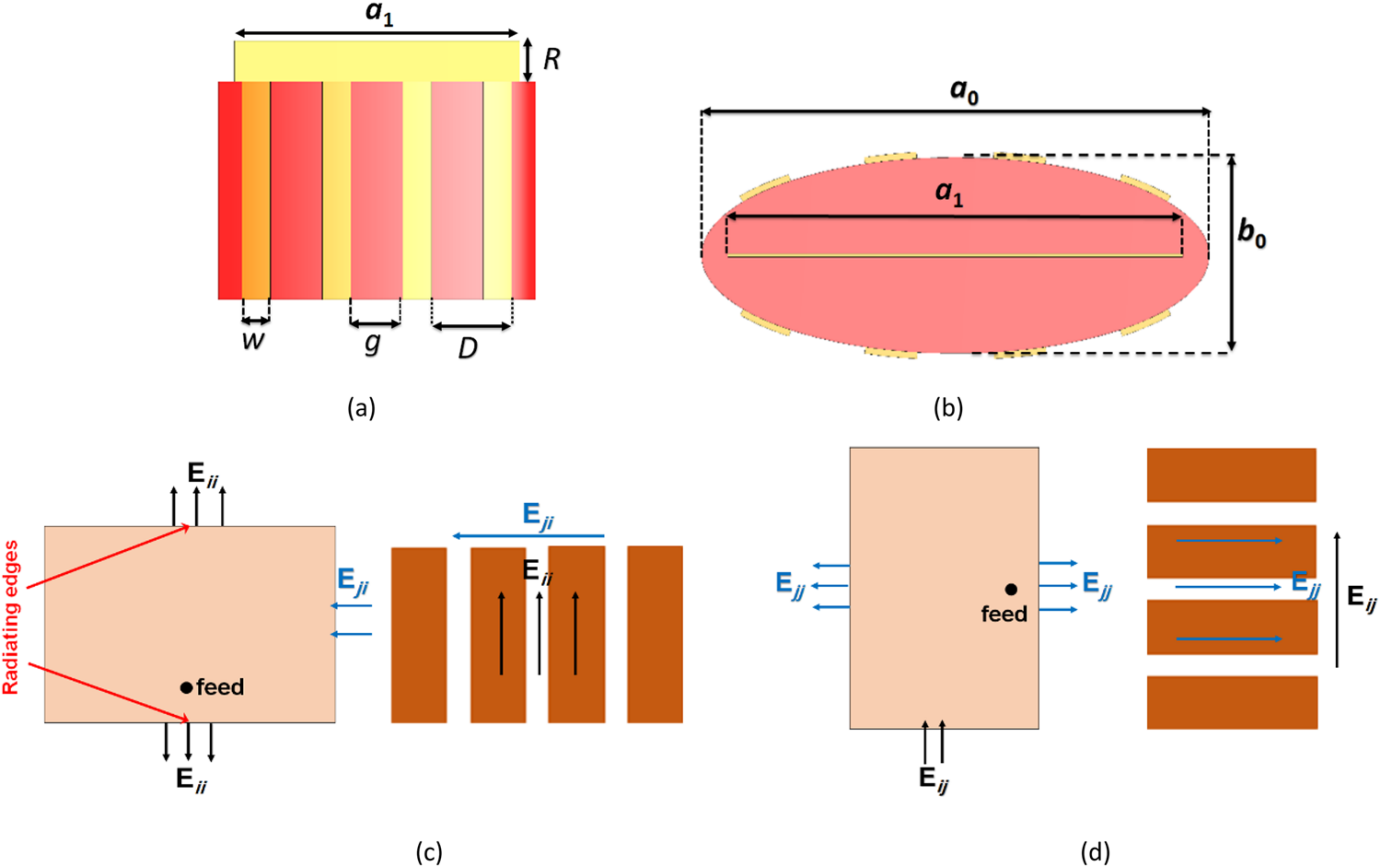
(**a**) Front view and (**b**) side view of the mantle cloak and metasurface strips on the patch antenna. Operating and orthogonal polarizations and the electric fields direction of the (**c**) ith patch and its own cloak strips, (**d**) jth patch and its own cloak strips.

Here, $a_{1}$ is equal to the patch width (*w*_1_) and $b_{1}$ is the patch thickness, which tends to zero (thin metallization). Based on the required values of $X_{opt}$, we need to consider capacitive metasurfaces to obtain negative surface reactances. In order to obtain the required surface reactance of Eq. (13), we use 1D conformal sub-wavelength metallic metasurface elements, such as parallel strips shown in Fig. 3a,b. The surface reactance of the conformal metasurface elements at frequency $\omega$ is obtained using an analytical grid-impedance expression given in^47,48^ as follows:

15 $$X_{s} = - \frac{{\eta_{0} \, c\pi }}{{\omega (\varepsilon_{r1} + 1)D}} \cdot \frac{1}{{Ln \, \csc \, \left( {\frac{\pi g}{{2D}}} \right)}}$$

where $\eta_{0}$ is the free space wave impedance, $c$ is the speed of light in free space, $\varepsilon_{r1}$ is the relative permittivity of the dielectric material, and $g$ is the spacing between the strips of the cloak. $D$ and $w$ represent grid parameters, the periodicity and width of the cloak strips, respectively.

The strips are anisotropic and the cloak has different “$X_{s}$”s for orthogonal and operating polarizations. For the orthogonal polarization, the $X_{s}$ satisfies Eq. (15), while for the operating polarization of the antenna (E-filed is along the strips of the cloak) shows a completely different value according to Eq. (16)^47,48^.

16 $$X_{S} = \frac{{\omega \eta_{0} D}}{2c\pi }\ln \csc (\frac{\pi w}{{2D}})$$

The operating polarization of the covered patch and the E-field direction on the cloak strips are shown in Fig. 3c,d. E_*ii*_ is the intrinsic electric field of ith element, E_*ij*_ is the coupled E-field form the ith element to the jth element, E_*ji*_ is the coupled E-field form the jth element to the ith element and E_*jj*_ is the intrinsic electric field of jth element. When the ith patch is radiating, its electric field (E_*ij*_) is perpendicular to the strips of the jth patch cloak and the desired $X_{s}$ (for the orthogonal polarization) is achieved. But, when jth patch is radiating, its electric field (E_*jj*_) is parallel to its own cloak strips and a different value is achieved for $X_{S}$(for the operating polarization).

**Figure 4:**
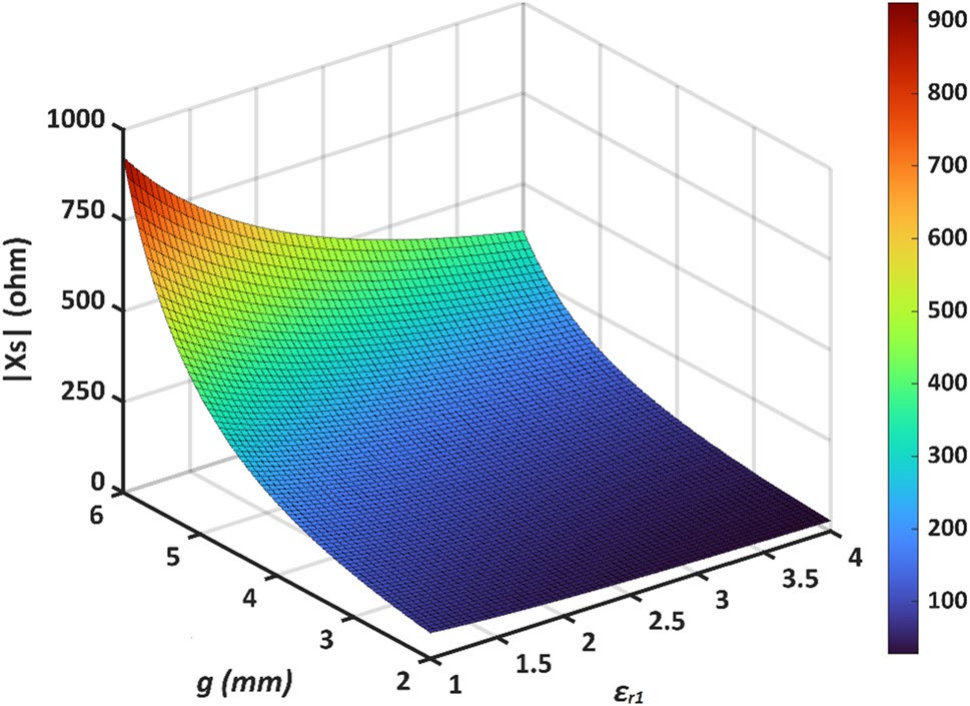
Variations of $X_{S}$ vs different values of *g* and *ε*_*r*1_.

By tuning the parameters of the cloak ($D$, $g$, and $\varepsilon_{r1}$), the required surface reactance can be obtained. It is worth noting that $D$ must be chosen as a fraction of the ellipse perimeter to obtain an integer number of strips. By choosing 8 strips, for example, the appropriate values for $g$ and $\varepsilon_{r1}$ are obtained. The surface reactance of conformal metasurface ($X_{s}$) vs different values of $g$ and $\varepsilon_{r1}$ at the operating frequency of 3.7 GHz is plotted in Fig. 4.

According to Eq. (15) and Fig. 4, it is obvious that the cloak structure acts as a perfect capacitor, where the capacitance increases with the increase of *ε*_*r*1_ and the decrease of *g*.

For the proposed structure, in the operating polarization, $X_{s}$ is − 258 Ω while for the orthogonal polarization, it is 23.42 Ω which represents a very low inductance (L = 1 nH) and, as a result, the cloak is almost transparent to the antenna itself.

The cloak strips covering the jth patch are not directly fed, but radiation from the adjacent ith patch induces a surface current on those strips as well as the jth patch. The induced current on the strips is anti-phase with the unwanted current induced on the patch. Thus, the undesired scattering is eliminated and the coupling between the ith and jth patches is reduced.

The strips of the cloak should only generate the aforementioned anti-phase current and should not resonate at the operating frequency of the antennas. If the length of the strips is equal to the patch length (near λ/2), they will start to resonate and degrade the antenna performance. To prevent these spurious resonances, the length of the strips is chosen slightly smaller than the length of the patch. This length difference is represented by “$R$” in Fig. 3a. Since the elements of the two arrays are identical, their mantle cloaks are the same as well, just rotated 90° relative to each other.

In the present work, since the cloak is almost transparent to the antenna itself at its operating polarization, the patch does not “see” its own cloak and it acts as a simple patch printed on a grounded substrate. Therefore, it is required to cover only the metal patch with the cloak, not the entire structure including the substrate and the ground plane. This has been shown and proven in^49,50^ for printed monopoles that have ground and substrate as well. It is worth noting that by inserting the cloak into the substrate, the thickness of the cloaked structure is minimized.

To summarize the design procedure, we present an overview below. The diameters of the elliptic cylinder, dielectric material, gap size, and periodicity of the strips are determined while considering the constraints of 3D printing. These dimensions are combined to maximize isolation:

1. *a*_1_ = *w*_1_ is the resonant length of the patch antenna at the operating frequency, and *b*_1_ = 0 is the patch thickness.
2. Maximum value for *b*_0_/2 is limited by the substrate thickness which is h = 1.524 mm; on the other hand, if *b*_0_ is too small, *a*_0_ would be unacceptably long according to Eq. (14). Therefore, *b*_0_ = 2.8 mm was selected and *a*_0_ is calculated from Eq. (14).

By placing these parameters in Eq. (13), $X_{opt}$ = − 258 Ω is obtained. Analytical grid-impedance ($X_{s}$) of Eq. (15) is set to be equal to $X_{opt}$.

3. By choosing the number of the cloak strips to be 8, the periodicity of the strips (*D*) is obtained by dividing the perimeter of the ellipse into this number. *ε*_*r*1_ according to 3D printing material is 2.2. Having *D* and *ε*_*r*1_, the remaining parameter of Eq. (15), *g*, is calculated to obtain the desired $X_{s}$ = − 258 Ω.
4. *R* is chosen so that the resonant frequency of the metasurface strips does not overlap with that of the patch antennas, and its appropriate value is obtained by optimization.

**Figure 5:**
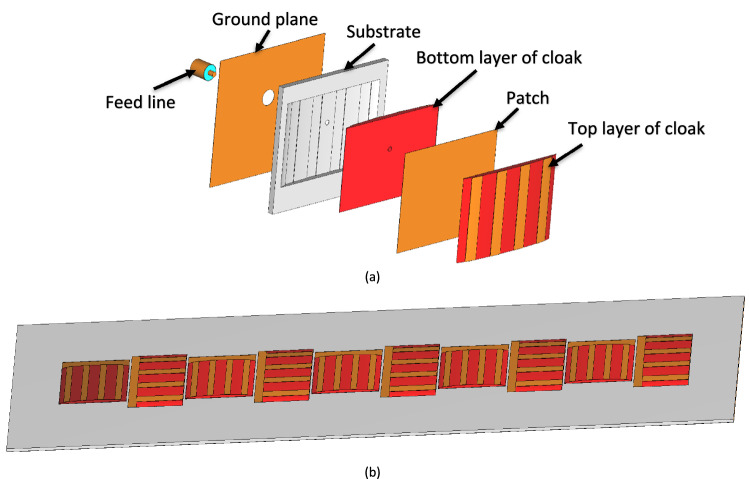
(**a**) Different layers of the mantle cloaked patch antenna, (**b**) 3D view of the two co-frequency interleaved cloaked arrays.

The different layers of the cloaked single patch antenna are shown in Fig. 5a and the two co-frequency interleaved cloaked arrays are illustrated in Fig. 5b. The layers of the cloak have metallic strips printed on thin dielectric layers (red), followed by a metal patch sandwiched between the identical top and bottom layers of the cloak. Then, the patch and the cloaking structure are laid on a grounded substrate layer (white) and a coaxial feed through is used that is connected to the ground plane. The optimized dimensions of the mantle cloaked patches are summarized in Table 2.

**Table 2:** Design parameters of the mantle cloaked patch antenna.

Parameters	*ε*_*r*1_	$g$	$D$	$a_{0}$	$b_{0}$	$a_{1}$	$b_{1}$	$R$
Value (mm)	2.2	5.1	7.9	31.22	2.8	31.1	0	4

## Results

### Simulation results

**Figure 6:**
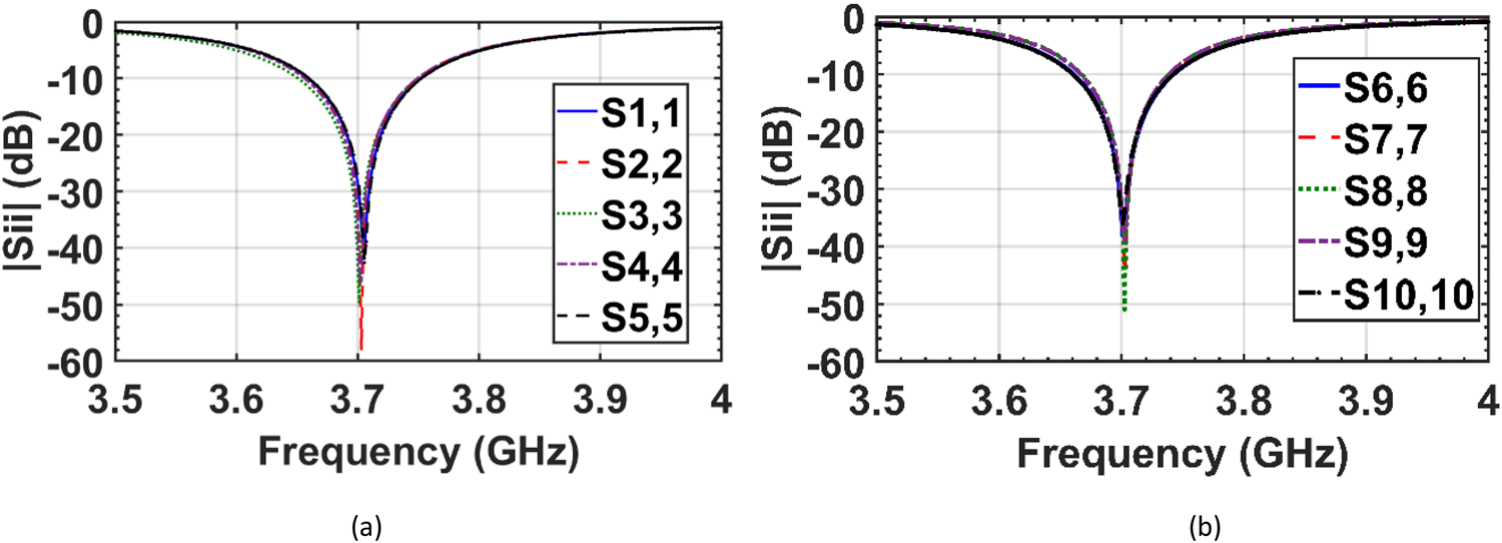
Simulated reflection coefficients of elements of the (**a**) isolated array I, and (**b**) isolated array II.

**Figure 7:**
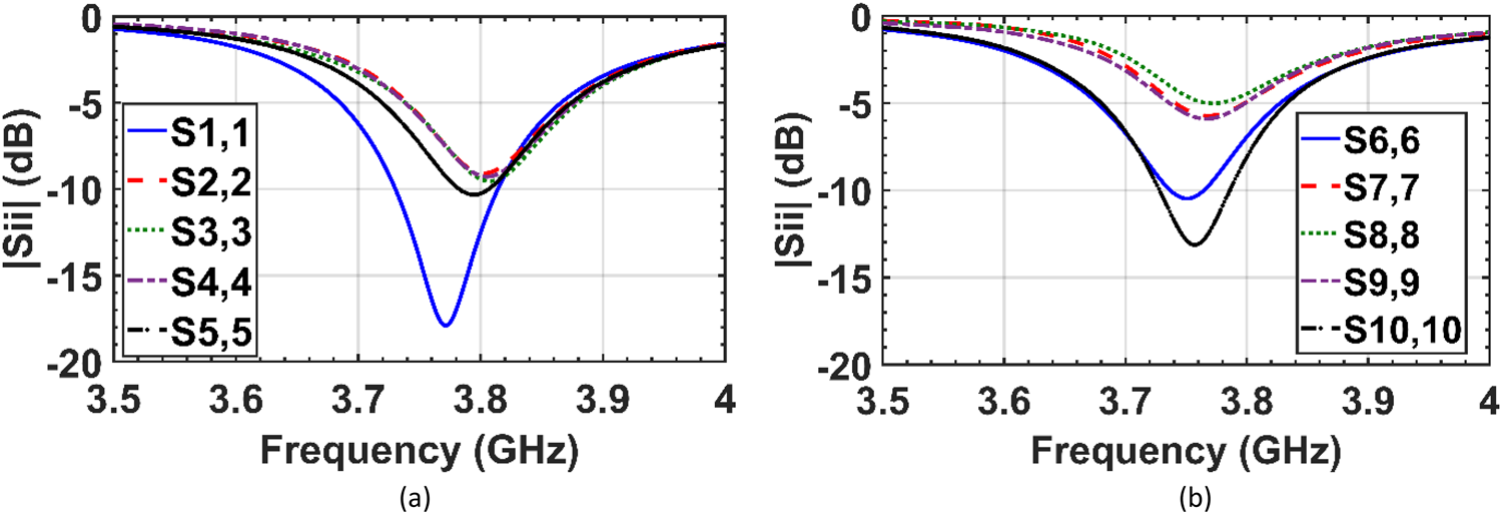
Simulated reflection coefficients of the interleaved elements of the (**a**) uncloaked array I, and (**b**) uncloaked array II.

The two co-frequency patch antenna arrays in the isolated case (Fig. 1a,b), uncloaked interleaved arrays (Fig. 1c), and cloaked arrays (Fig. 5b) are simulated using the 3D full-wave solver of CST Microwave Studio. The reflection coefficients of the elements of the two isolated (individual) arrays and interleaved uncloaked arrays are illustrated in Figs. 6 and 7, respectively. As can be seen in Fig. 7, due to the strong coupling between the tightly spaced elements of the two arrays, the input impedances of the antennas in the uncloaked configuration are changed and degraded their reflection coefficients.

**Figure 8:**
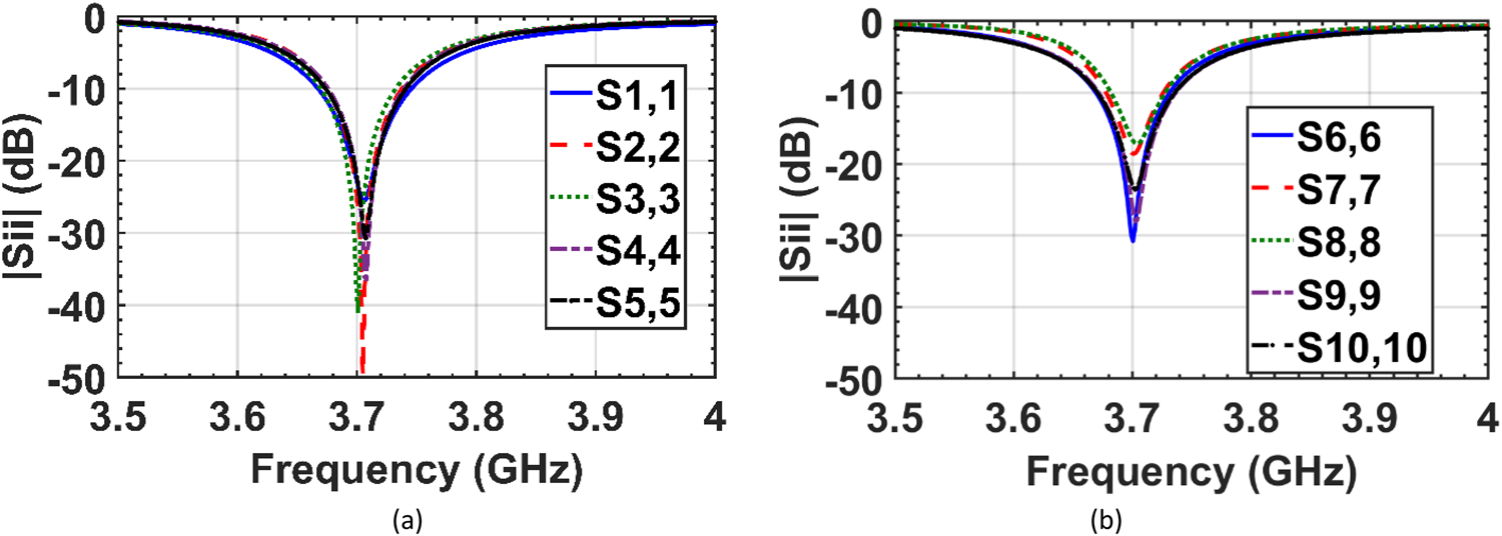
Simulated reflection coefficients of the interleaved elements of the (**a**) cloaked array I, and (**b**) cloaked array II.

The reflection coefficients of the elements of the two interleaved cloaked arrays (Fig. 5b) are shown in Fig. 8. This figure indicates that the impedance matching has been retrieved for both arrays as the results of the cloaked arrays are similar to the isolated case shown in Fig. 6.

### Measurement results

**Figure 9:**
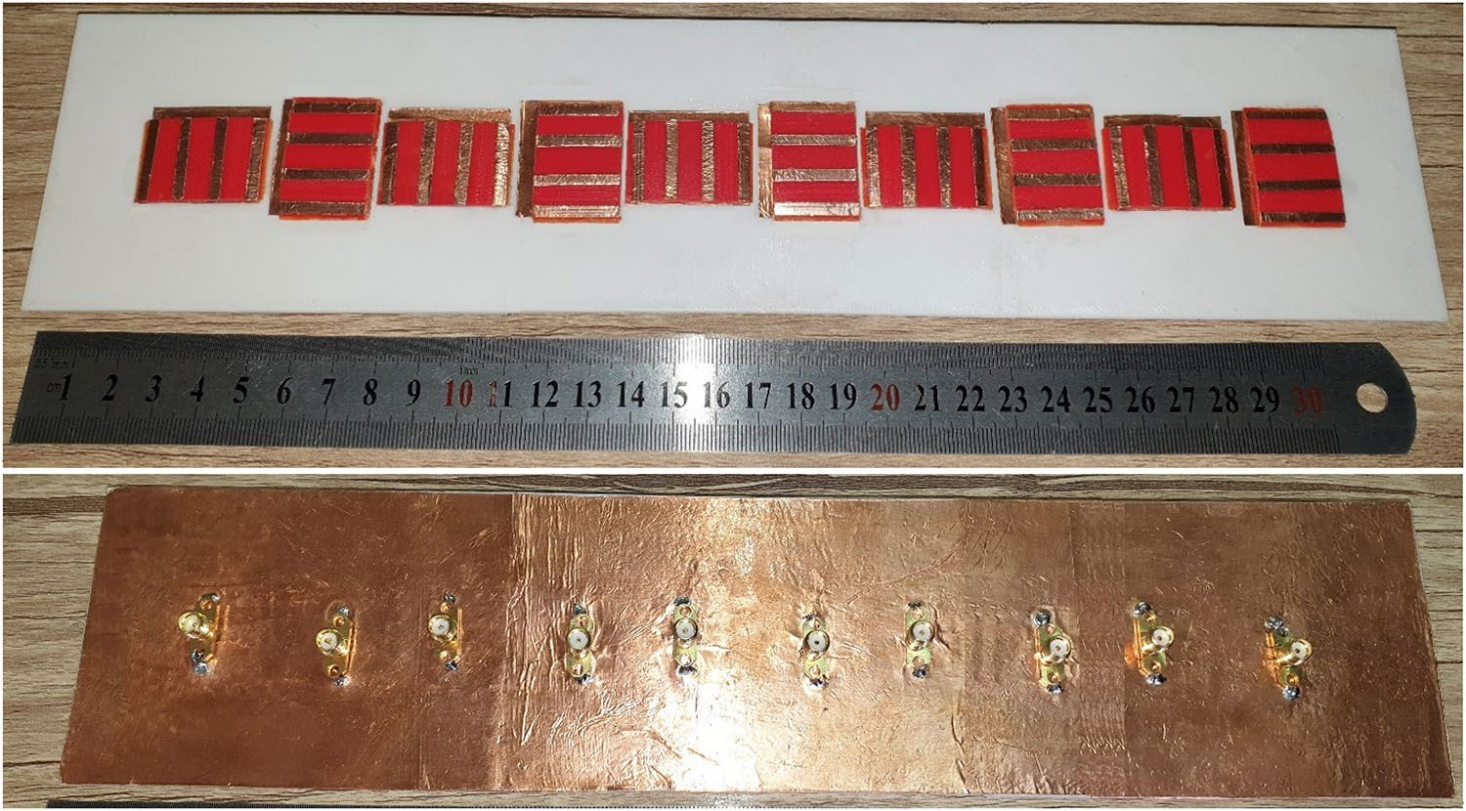
Front and back views of the fabricated two cloaked interleaved arrays.

**Figure 10:**
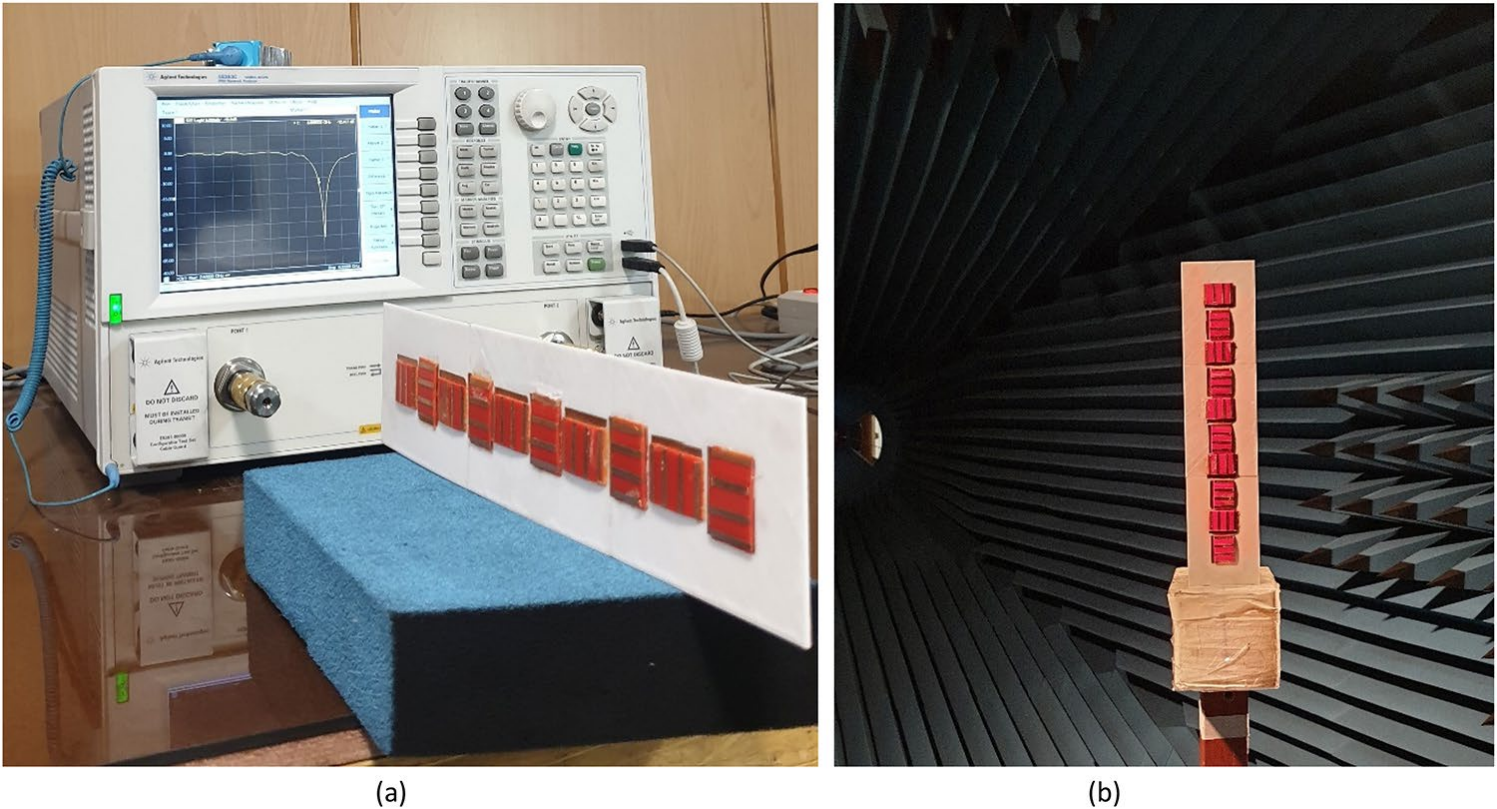
Measurement setup for (**a**) reflection coefficients, (**b**) radiation patterns.

To validate the performance of the proposed cloaking structure, the cloaked arrays are fabricated with 3D printing technology and is illustrated in Fig. 9. The setup of Fig. 10 is used to measure the reflection coefficient and radiation performance of the antennas.

**Figure 11:**
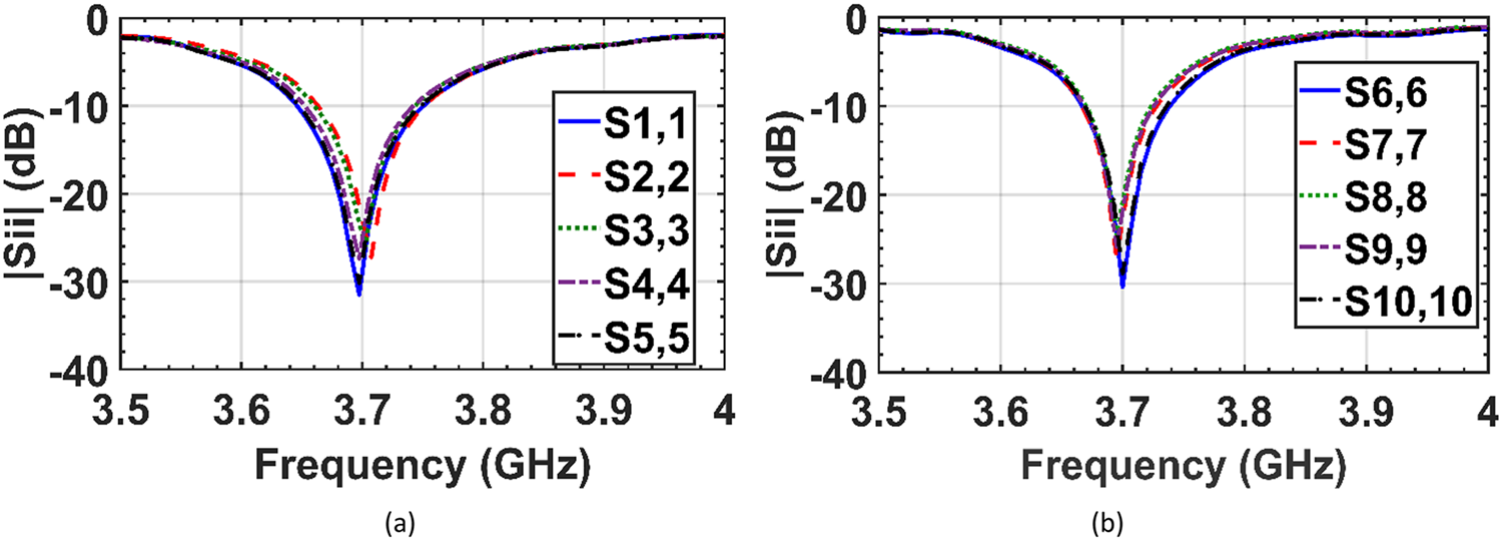
Measured reflection coefficients of the elements of the (**a**) cloaked array I, and (**b**) cloaked array II.

The reflection coefficients of the fabricated cloaked arrays are measured and shown in Fig. 11. Good agreement between measured and simulated results has been demonstrated.

### Mutual coupling between the elements

**Figure 12:**
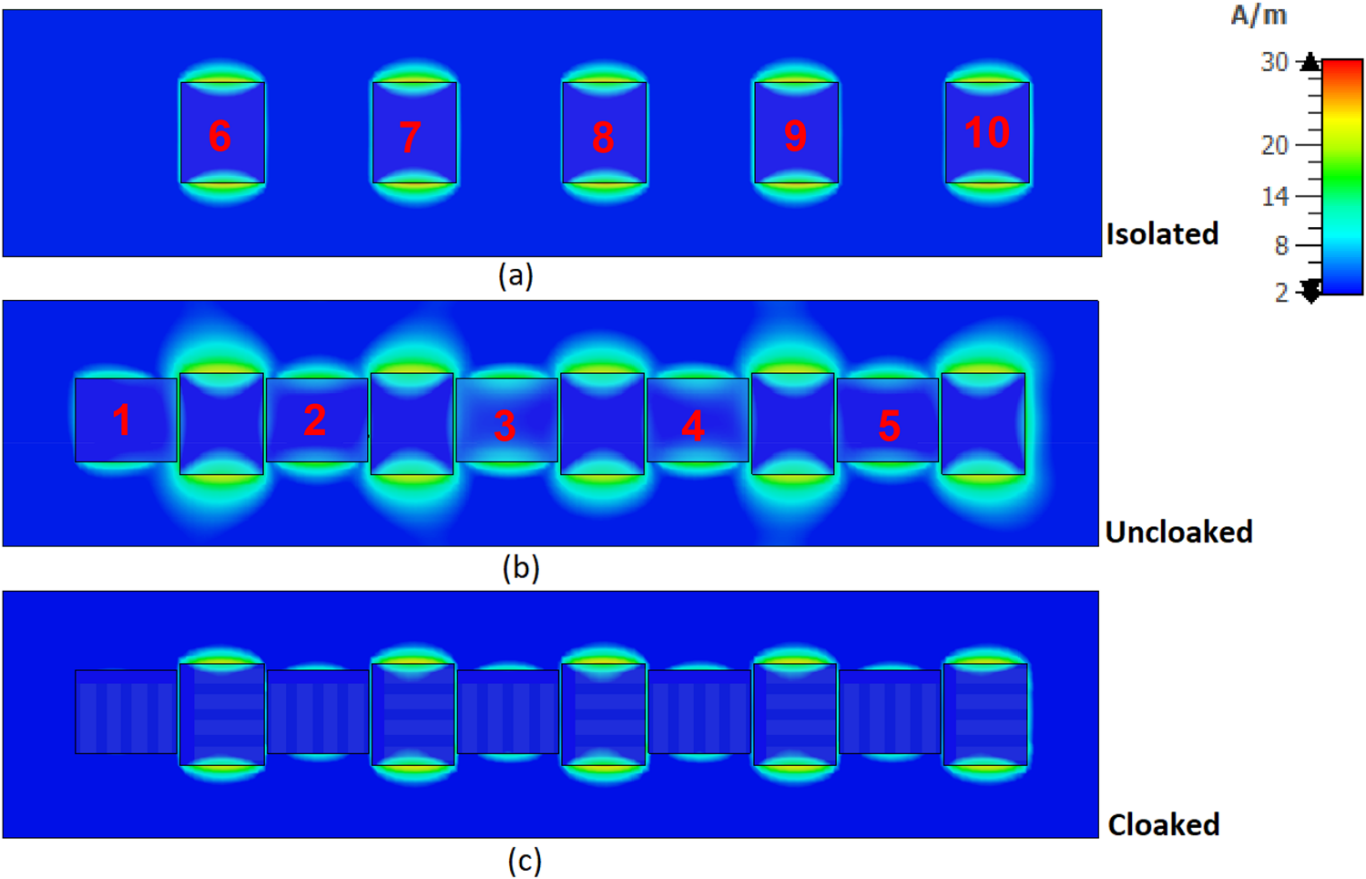
Surface currents of the elements of the (**a**) array II when isolated, (**b**) two interleaved uncloaked arrays when array II is excited and array I is matched, (**c**) two interleaved cloaked arrays when array II is excited and array I is matched.

Figure 12a–c shows the surface current on the elements of two arrays in different cases. The elements of array II (#6–10) are excited and they induce some surface currents on the matched (unexcited) elements of array I (#1–5). These unwanted induced surface currents on the elements of array I can be canceled by the anti-phase surface current generated by the cloak structure. Figure 12a shows the excited array II when isolated. Figure 12b,c show the two interleaved uncloaked and cloaked arrays, respectively, when the elements of the array II are excited and those of the array I are matched. As it can be seen in Fig. 12c, the anti-phase current of the cloak weakens the coupling between two arrays and cancels the unwanted induced surface current on elements of array I due to the radiation of array II. Similar behavior is observed when array I is excited and array II is matched, which is not shown here for brevity.

When two arrays are excited simultaneously, there are both intrinsic currents (i.e. currents coming from the power supply) and induced currents on the elements. Since the intrinsic currents are stronger than the induced currents, the reduction of the induced currents due to the presence of the cloak is not distinguishable. But when exciting only one array, the reduction in the induced currents is more clear.

**Figure 13:**
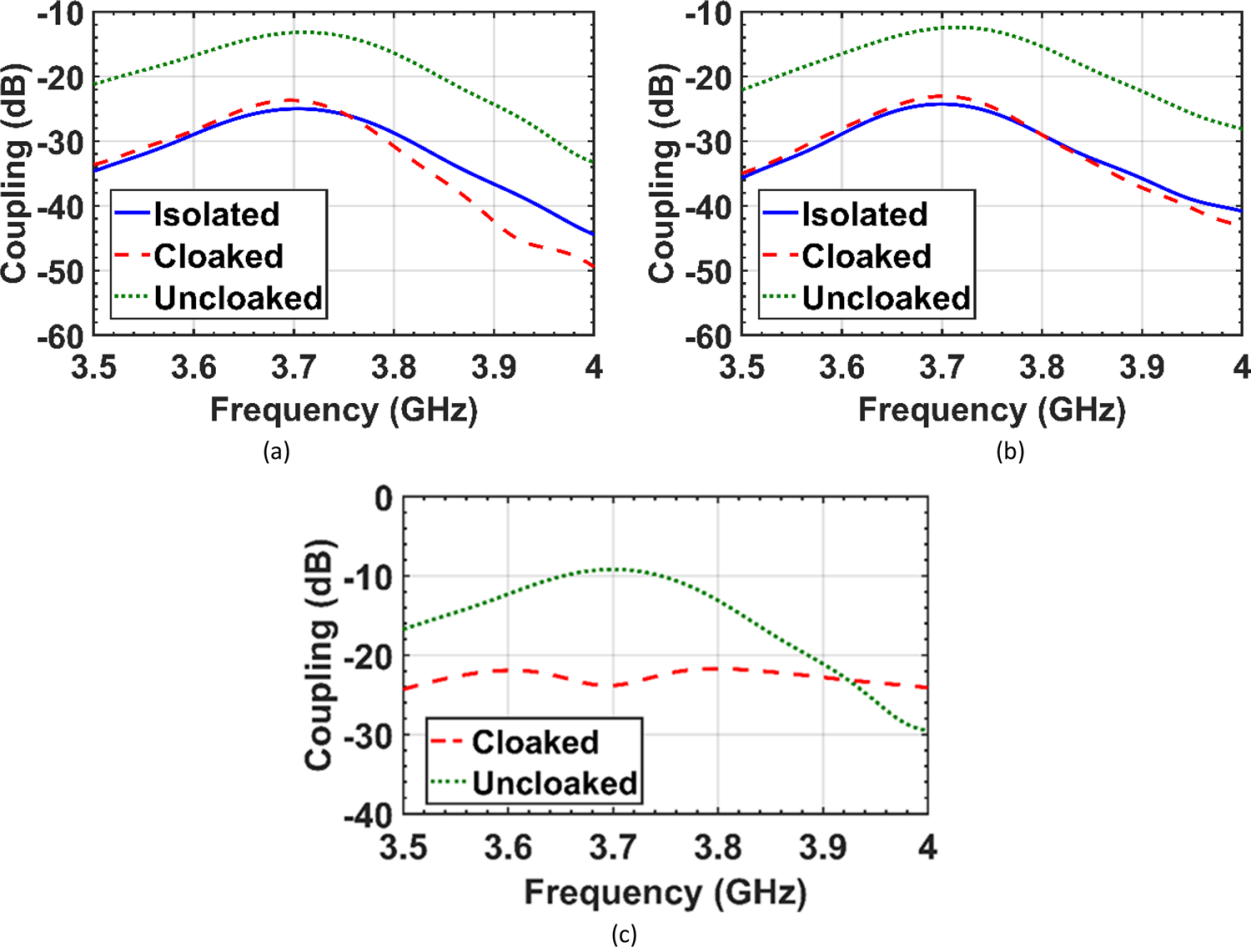
Simulated mutual coupling between the central elements of the two arrays in the isolated, uncloaked and cloaked configurations. (a) coupling between the elements of the array I, S_23_, (b) coupling between the elements of array II, S_78_, (**c**) cross-coupling between the adjacent elements of the two interleaved arrays, S_38_.

The mutual coupling between the central elements of the two arrays in the isolated, uncloaked and cloaked configurations is shown in Fig. 13 for comparison. As expected, in the tightly spaced interleaved uncloaked arrays, a strong cross-coupling between the elements impacts the input impedances of the antennas and causes significant mismatch (drop by 15 dB). In Fig. 13a,b, upon cloaked arrays I and II, significant improvement in decoupling between the elements of the same array is observed. Figure 13c compares the cross-coupling between the neighboring elements of arrays I and II before and after cloaking. For brevity, only the coupling between the closest elements, and thus with the highest coupling, is compared in the isolated, uncloaked and cloaked cases. In the cloaked configuration, the isolation between the nearest elements is improved by at least 10 dB compared to the uncloaked case. Since the inter-element spacing of “each” array is large enough (d = 0.7λ_0_), the coupling between non-adjacent elements of each array (S_13_, S_24_, …) is very low in all cases.

### Radiation characteristics

**Figure 14:**
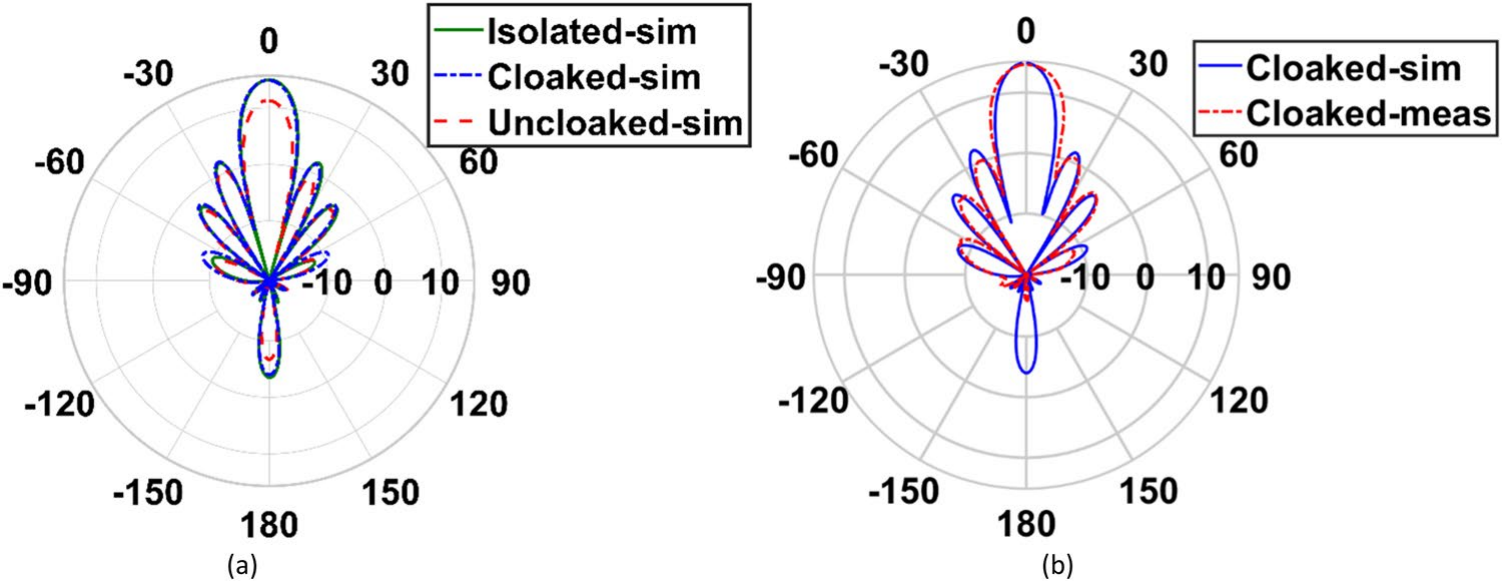
(**a**) Comparison between the gain pattern of array I in isolated, cloaked and uncloaked cases, (**b**) measured and simulated patterns of the cloaked case.

**Figure 15:**
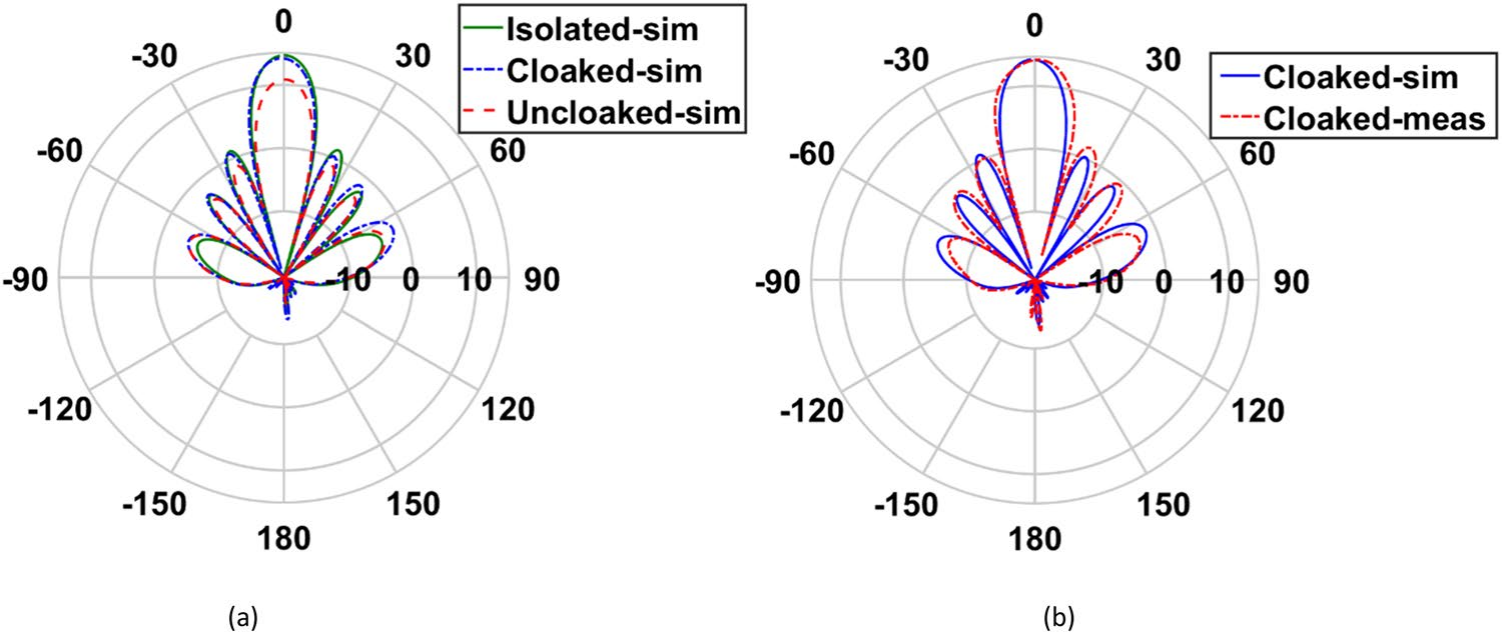
(**a**) Comparison between the gain pattern of array II in isolated, cloaked and uncloaked cases, (**b**) measured and simulated patterns of the cloaked case.

In this section, we compare the radiation characteristics of both arrays in three investigated cases. A 2D polar plots of the gain patterns of simulated isolated, uncloaked and cloaked cases are compared with the measured cloaked case for arrays I and II, and are shown in Figs. 14 and 15.

**Figure 16:**
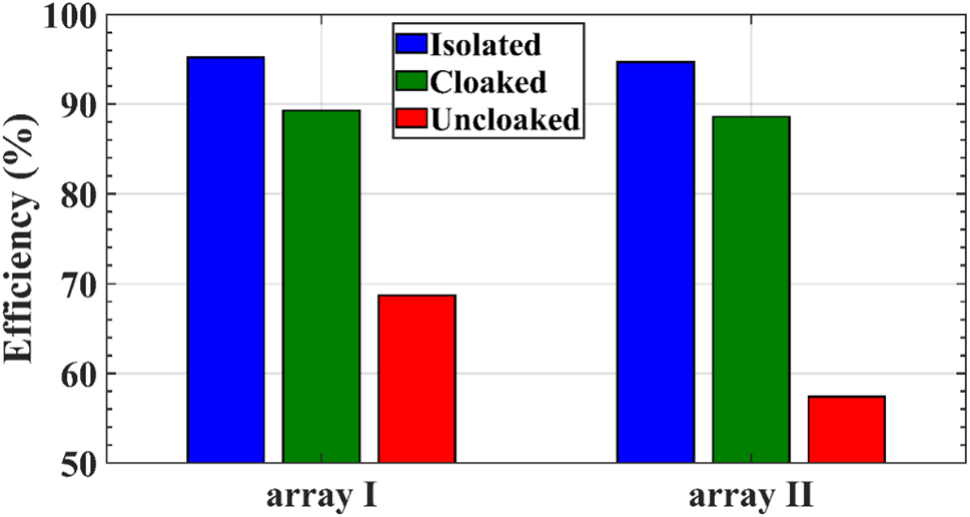
The total efficiency of the arrays I and II for three investigated cases.

In the array configuration, the elements of two arrays are placed very close to each other, therefore the total efficiency of both arrays are expected to be degraded due to the strong coupling between the elements in the interleaved uncloaked configuration. The total efficiency of the arrays in isolated, interleaved uncloaked and cloaked configurations are compared in Fig. 16. The total efficiency indicates the ratio of the radiated power (*P*_*radiated*_) to the input power (*P*_in_) of the antenna, and we have:

17 $$Total \, Efficiency \, (e_{T} ) = \frac{{P_{radiated} }}{{P_{in} }}$$

18 $$P_{in} = P_{radiated} + P_{ohmic} + P_{dielectric} + P_{surface \, waves} + P_{reflected}$$

*P*_*reflected*_ is proportional to the input reflection coefficient (*S*_11_) of the antenna:

19 $$P_{reflected} \propto \left| {S_{11} } \right|^{2} = \left| { \frac{{Z_{in} - Z_{0} }}{{Z_{in} + Z_{0} }}} \right|^{2}$$

As the mutual coupling is predominantly strong in the closely spaced antennas, this can deteriorate the input impedance of each radiating element in the array and adversely affect the radiation efficiency of the array. Ohmic, dielectric and surface waves losses are almost the same for the cloaked and uncloaked antennas, but since the return loss of the antenna is high in the uncloaked case, it significantly reduces the total efficiency compared to the cloaked and isolated cases.

Since the elements of array II are arranged in the E-plane, that is, along the direction of their radiating edges, the mutual coupling between the elements of this array has more unfavorable effects on its total efficiency in the uncloaked case than that of array I.

## Discussion

In the cloaking of passive structures, the success of the cloaking structure in reducing the scattering is shown by comparing the cloaked and uncloaked cases, ensuring in the cloaked case, the radiated fields should not be perturbed. In this work, since the cloak covers an active antenna, the ultimate goal is to retrieve the radiation characteristics of the antenna similar to the isolated case. Therefore, the criterion for evaluating the performance of the cloak is to compare the cloaked and isolated cases in terms of radiation characteristics and scattering parameters. Here, the return loss, radiation patterns, gain and efficiency of the isolated, cloaked and uncloaked cases are compared in Figs. 6, 7, 8, 9, 10, 11, 12, 13, 14, 15 and 16 that verify the success of the proposed method.

Decoupling between the antennas is mainly achieved using frequency-selective filters. Since in the proposed structure, two antenna arrays are working at the same frequency, it is not possible to decouple them using the filters. In the previous studies, two co-frequency and orthogonally polarized antennas are isolated by adding extra space between them using different techniques. Utilizing the cloak to decouple the patch antennas has not been studied before. In Table 3, a comparison is carried out between the previously published techniques and the present work on decoupling array elements in terms of the spacing between the elements, the complexity of the decoupling method, and the decoupling level (polarization isolation).

**Table 3:** Comparison between the present mantle cloaking method with other techniques used for decoupling the antennas.

References	Characteristics					
	Method of decoupling	Edge to edge element Spacing	Complexity of decoupling method	Decoupling level (pol. isolation) (dB)	Frequency (GHz)	Antenna type
^51^	Modified array antenna decoupling surface Defected ground structures	0.034 λ_0_	High with 3D structure and two-stage design	26	3.7	Patch
^52^	Ceramic superstrate-based decoupling method	0.28 λ_0_	Medium with 3D structure	25	3.5	Dipole
^53^	Central-symmetry decoupling technique	0.11 λ_0_	Low	22	2.5	Patch
^54^	Mixed radiation modes	0.11 λ_0_	Medium with 3D structure	23	2.5	Patch
^55^	Decoupling dielectric stubs	0.27 λ_0_	Medium with 3D structure	20	4.75	Patch
^56^	Frequency selective surface	0.22 λ_0_	Medium with 3D structure	18	4	Patch
^57^	Array-antenna decoupling surface	0.09 λ_0_	High with 3D complex structure	17	2.45	Patch
This work	Mantle cloaking	0.012 λ_0_	Low with an almost planar structure	24	3.7	Patch

The 1 mm (0.012λ_0_) spacing between the elements in the proposed configuration is much smaller than that of the previously published decoupled arrays. However, this spacing is used to avoid shortening between the patches and to consider the manufacturing constraints. Theoretically, this spacing can be reduced to almost zero since two adjacent patches do not “see” each other and are completely isolated from each other.

According to Figs. 6, 7 and 8, in the interleaved uncloaked case the resonant frequency of the patch elements is shifted from the desired frequency (3.7 GHz), and the reflection coefficients of some elements are significantly degraded due to the change in input impedance as a result of the strong coupling between the elements. Resonant frequencies were retrieved in the cloaked case and the cloaked antennas have the same performance as the isolated case. Measurement results in Fig. 11 validate the simulation and confirm that adjacent antennas do not have pronounced coupling to each other after cloaking. Figure 13 shows similar coupling levels in the cloaked and isolated cases, where cloaking provided at least 13 dB coupling reduction compared to the uncloaked case at the resonant frequency.

According to Figs. 14 and 15, in the interleaved uncloaked case, the strong coupling between the adjacent elements of the arrays perturbs the constructive far-field summation (array gain), thus the broadside gain decreases and the sidelobe level (SLL) increases. As previously discussed, since the elements of array II are arranged in the E-plane, the mutual coupling between the elements of this array has more adverse effects on its efficiency in the uncloaked case compared to that of array I, as shown in Fig. 16. The radiation patterns of both arrays in the cloaked case are the same as those in the isolated case with no significant deviation. The results are validated by measurements. The gain, SLL and efficiency of the two arrays in the three investigated cases, along with the measurement results for the cloaked case are summarized in Table 4.

**Table 4:** Comparison between the radiation characteristics of the arrays in the isolated, uncloaked and cloaked cases.

Case	Isolated	Uncloaked	Cloaked	
			Simulation	Measurement
Array I				
Gain (dB)	14.95	12.3	14.88	14.64
SLL (dB)	12.9	10.4	12.7	13.1
Efficiency (%)	95.2	68.7	89.3	–
Array II				
Gain (dB)	14.7	11.87	14.22	14.19
SLL (dB)	13.4	12	13.3	12.3
Efficiency (%)	94.7	57.4	88.6	–

As it can be seen, by covering two densely spaced interleaved patch arrays with tailored elliptical mantle cloaks comprising of vertical metasurface strips on a dielectric elliptic cylinder substrate, their input impedance, isolation, and radiation characteristics are retrieved similar to those of an isolated environment. An edge-to-edge spacing as small as 1 mm (*λ*_0_/80) between the patches and decoupling of co-frequency arrays is achieved using the scattering cancellation method and elliptical mantle cloaks, which is not possible with frequency-sensitive filters. The cloak structure can also be designed using more complex metasurfaces^58,59^ to achieve alternative applications.

The main novelty of this work is the possibility to bypass the intrinsic limitation of passive cloaking using two orthogonally-polarized antennas and anisotropic mantle cloaks. As almost the same real-estate, which is typically used for only one array, can now be utilized for interleaving two arrays with orthogonal polarizations, potentially leading to significant space savings in practical applications calling for compact designs.

## Conclusion

In this work, for the first time, the concept of mantle cloaking using the tailored elliptical metasurfaces is proposed for polarization decoupling of two densely packed linear patch arrays working at the same frequency. The simulation and measurement results demonstrate that by covering the elements of the two interleaved arrays with appropriate cloaks, not only the undesired mutual cross-coupling effects are eliminated, but also the radiation characteristics of the antennas are restored, resembling those of isolated arrays. The measurement results validate the simulated performance of the design. This design is suitable for implementing tightly spaced antenna arrays for dual polarization communication applications, especially for compact and portable 5G and 6G cell phones.

## Data Availability

The datasets generated during and/or analysed during the current study are available from the corresponding author on reasonable request.
